# Pre-conception clinical risk factors differ between spontaneous and indicated preterm birth in a densely phenotyped EHR cohort

**DOI:** 10.1186/s12884-025-07166-2

**Published:** 2025-02-12

**Authors:** Jean M. Costello, Hannah Takasuka, Jacquelyn Roger, Ophelia Yin, Alice Tang, Tomiko Oskotsky, Marina Sirota, John A. Capra

**Affiliations:** 1 Bakar Computational Health Sciences Institute, UCSF, San Francisco, USA; 2 Department of Pediatrics, UCSF, San Francisco, USA; 3 Graduate Program in Oral and Craniofacial Sciences, UCSF, San Francisco, USA; 4 Graduate Program in Biological and Medical Informatics, UCSF, San Francisco, USA; 5 Department of Obstetrics, Gynecology & Reproductive Sciences, UCSF, San Francisco, USA; 6 Graduate Program in Bioengineering, UCSF and UC Berkeley, San Francisco and Berkeley, USA; 7 Department of Epidemiology and Biostatistics, UCSF, San Francisco, USA

**Keywords:** Spontaneous preterm birth, Indicated preterm birth, Electronic health records, Diagnosis associations

## Abstract

**Background:**

Preterm birth (PTB) is the leading cause of infant mortality. Risk for PTB is influenced by multiple biological pathways, many of which are poorly understood. Some PTBs result from medically indicated labor following complications from hypertension and/or diabetes, while many others are spontaneous with unknown causes. Previously, investigation of potential risk factors has been limited by a lack of data on maternal medical history and the difficulty of classifying PTBs as indicated or spontaneous. Here, we leverage electronic health record (EHR) data (patient health information including demographics, diagnoses, and medications) and a supplemental curated pregnancy database to overcome these limitations. Novel associations may provide new insight into the pathophysiology of PTB as well as help identify individuals who would be at risk of PTB.

**Methods:**

We quantified associations between maternal diagnoses and preterm birth both with and without controlling for maternal age and socioeconomic factors within a University of California, San Francisco (UCSF), EHR cohort with 10,643 births (*n*_*term*_ = 9692, *n*_*spontaneous_preterm*_ = 449, *n*_*indicated_preterm*_ = 418) and maternal pre-conception diagnoses derived from International Classification of Diseases (ICD) 9 and 10 codes.

**Results:**

Thirty diagnoses significantly and robustly (False Discovery Rate (FDR) < 0.05) associated with indicated PTBs compared to term. We discovered known (hypertension, diabetes, and chronic kidney disease) and less established (blood, cardiac, gynecological, and liver diagnoses) associations. Essential hypertension had the most significant association with indicated PTB (adjusted p_BH_ = 4 × 10^–20^, adjusted OR = 6 (95% CI 4-8)), and the odds ratios for the significant diagnoses ranged from 2 to 23. The results for indicated PTB largely recapitulated the diagnosis associations with all PTBs. However, no diagnosis significantly associated with spontaneous PTB.

**Conclusions:**

Our study underscores the limitations of approaches that combine indicated and spontaneous births. When combined, significant associations were almost entirely driven by indicated PTBs, although the spontaneous and indicated groups were of a similar size. Investigating the spontaneous population has the potential to reveal new pathways and understanding of the heterogeneity of PTB.

**Supplementary Information:**

The online version contains supplementary material available at 10.1186/s12884-025-07166-2.

## Background

Preterm birth (PTB) is the leading cause of infant mortality worldwide [1] and can result in serious acute and long-term health consequences [2, 3]. There are multiple proposed pathways for preterm birth, but its etiology remains poorly understood [4–7]. About two thirds of PTBs in the US are classified as spontaneous preterm while the remaining third are medically indicated (iatrogenic) preterm [8]. An indicated preterm birth is typically initiated based on a list of risk factors, which includes preeclampsia, diabetes complications, intrauterine abnormalities, and placental abnormalities [9]. Maternal risk factors for indicated preterm birth include older maternal age, heart disease, hypertension, diabetes, tobacco use, previous preterm delivery, and socioeconomic factors [8, 10]. Some of these risk factors, such as poorly managed hypertension, may be present prior to pregnancy. Spontaneous preterm birth, by contrast, lacks a defined set of known risk factors, and the pathophysiology behind it remains poorly understood [8].

Several maternal risk factors for spontaneous preterm birth have been proposed, including prior spontaneous preterm birth, gynecological anatomy variation, short inter-pregnancy interval, and multiple gestations [10]. Prior spontaneous preterm birth is the strongest known risk factor. In the United States, racism is a risk factor for spontaneous preterm birth [11], with higher rates among non-Hispanic Black individuals when compared to white individuals, including after adjustment for socioeconomic variables [12]. Some studies have explored whether gene–gene and/or gene-environment interactions might exist to explain racial disparities, but these studies are limited to cohorts of a few hundred patients [10].

Improved understanding of pathways and clinical factors leading to preterm birth could lead to better interventions to prevent preterm birth, especially spontaneous preterm birth. Investigating pre-pregnancy diagnoses associated with subsequent PTB has the potential to generate hypotheses about pathways towards PTB. Many large studies of conditions associated with PTB rely on registry data, which provides limited diagnosis information [13, 14]. In contrast, EHR databases provide dense phenotyping including demographics, diagnoses, and medications over time that can provide insights difficult to obtain from other data sources. However, EHR systems may not distinguish between spontaneous and indicated deliveries [15, 16]. Nonetheless, EHR data are particularly well-suited to the study of pregnancy [17]. For instance, machine learning models have used EHR data to accurately predict preterm birth in thousands of patients [16]. While complex machine learning models have great potential to improve obstetric and gynecological care, novel insights from straightforward methods applied to EHR data could more easily translate to pathway discovery and evidence-based care.

In this study, we explore the potential of an EHR system combined with a curated delivery database to validate known associations and identify novel associations between pre-conception diagnoses and spontaneous and indicated PTB. We expect that hypertension and diabetes will be strongly associated with indicated PTB but have weaker associations with spontaneous PTB. Additionally, we hope to find significant pre-conception spontaneous PTB risk factors that could later lead to the discovery of the currently unknown biological pathways preceding spontaneous PTB.

With our approach, we reproduce widely known preterm birth risk factors including major chronic diseases. Moreover, we discover several new preterm birth associations with less-studied diagnoses such as decreased white blood count. We also demonstrate that all significant associations with PTB are driven by indicated PTBs and that no diagnoses significantly associate with spontaneous preterm birth.

## Methods

### Birth data

We identified births using a perinatal database (PDB), which is maintained and curated by obstetricians at UCSF. This database contains detailed information about each delivery that takes place in the hospital and includes whether the delivery was spontaneous or indicated. Newborn patient IDs in this database are linked to newborn patient IDs in the EHR. The start of pregnancy was determined by subtracting gestational weeks from the delivery date.

### Diagnosis data

Diagnosis information was obtained from UCSF’s Observational Medical Outcomes Partnership (OMOP) de-identified EHR database, using mapped newborn patient IDs from the PDB. To be considered, diagnoses must have an ICD-9 or ICD-10 code, map to a phecode, and have a start date prior to the start of pregnancy. Each diagnosis was considered as a binary variable (present vs absent), rather than a count. This was done so that chronic condition diagnoses (which may be recorded at multiple visits) would not overwhelm our results. Phecodes were truncated after the first decimal point to provide an appropriate level of detail to diagnoses (Figure S2).

### Selection criteria

**Fig. 1:**
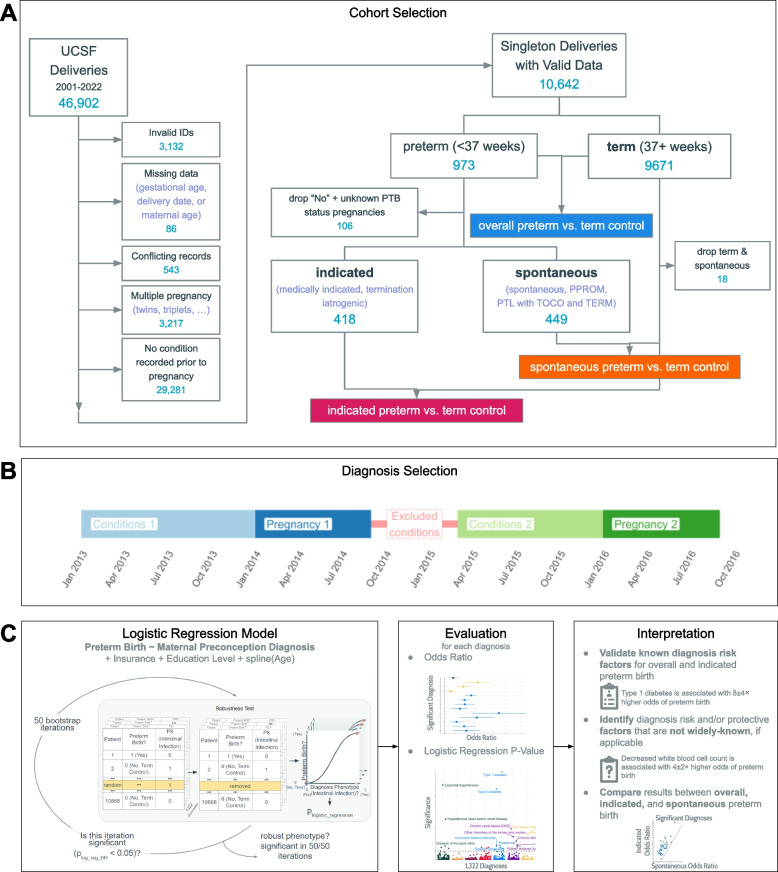
Schematic of the Approach for Testing Associations Between Preterm Birth and Diverse Diagnoses. **A** Criteria for identifying the 10,642 individuals studied and assigning them to overall preterm, indicated preterm, spontaneous preterm, and term groups for subsequent logistic regression analyses. We also tested associations in a larger cohort of 39,896 individuals identified without requiring a diagnosis recorded before pregnancy (Supplementary Material). **B** Diagnoses before conception are used in this study. For a person’s first recorded birth (or only recorded birth), diagnoses are recorded from the start of their record until the start of conception. If multiple births are recorded for the same individual, diagnoses for subsequent births are recorded starting 6 months from after the previous delivery to the start of the next conception. **C** Overview of the logistic regression analysis, covariates, evaluation, and interpretation for associations between preterm birth and the 1322 diagnoses considered. *P*-values were adjusted for multiple hypothesis testing and a permutation test was used to ensure associations were robust

We selected our sample from all deliveries at UCSF between 2001 and 2022. To be included, PDB maternal patient IDs must map to maternal patient IDs in the UCSF EHR, and only one record per delivery may be present (Fig. 1a). Additionally, deliveries must be singleton, have a recorded gestational age, have a recorded delivery date, and be from an individual with at least one diagnosis prior to the start of pregnancy. To construct the cohort for our sensitivity analysis, we did not remove those who lacked a diagnosis prior to conception. All other cohort creation and analytic steps were performed in the same manner as the main analysis.

### Assigning diagnoses to pregnancies

For our main analysis, we included multiple deliveries from the same individual. Diagnoses were assigned per pregnancy in the following way: the diagnosis must be prior to the start of the pregnancy but not in the six-month period following the most recent delivery (Fig. 1b). If no prior delivery was recorded, then diagnoses at any time prior to the pregnancy were included.

### Spontaneous and indicated preterm definitions

A team of clinicians manually marked all 10,668 pregnancies in this cohort with a PTB status of “No” (this indicates a term birth), “spontaneous,” “PPROM,” “medically indicated,” “Termination Iatrogenic,” or “PTL with TOCO and TERM.” Additionally, pregnancies had data on gestational age, maternal age, maternal education level in years, and insurance type. Using the gestational age data, 975 of the newborns were delivered at fewer than 37 weeks, and 9,693 newborns were delivered at 37 weeks or more (Fig. 1a). Pregnancies that were labeled with a gestational age of less than 37 weeks and a PTB status of “medically indicated” or “Termination Iatrogenic” were classified as indicated. Pregnancies that were labeled with a gestational age of less than 37 weeks and a PTB status of “spontaneous,” “PPROM,” or “PTL with TOCO and TERM” were classified as spontaneous.

In some cases, the PTB status value did not align with the gestational age value. In these cases, the pregnancies were dropped. Pregnancies marked with a gestational age of 37 + weeks and a spontaneous PTB status (*n* = 18) represent individuals who experienced medically interrupted spontaneous preterm labor and delivered after term. It is unknown why some pregnancies would be marked with a gestational age less than 37 weeks and “No” PTB status (*n* = 106).

### Diagnosis-PTB association analysis

For all diagnoses occurring in at least one recorded birth, we applied adjusted logistic regression to test the associations between each diagnosis and unstratified PTB, indicated PTB, and spontaneous PTB. Odds ratios and *p*-values were calculated using the glm() function in R.

### Covariates

Based on previous findings regarding PTB, we wanted to adjust for maternal age and socioeconomic status (SES). We have maternal age as a variable, and we use insurance status and maternal education as proxies for SES. A smoothing spline was applied to maternal age to capture the non-linear relationship between age and PTB [32]​. Maternal education was reduced from the raw number of years value to categories: less than 12th grade, 12th grade, and college. As missingness for covariates was present in the data, “unknown” was considered to be a separate category for each.

### *P*-value significance, bootstrapping and plotting

When classifying a diagnosis as significant or not significant, *p*-values were adjusted for multiple hypothesis testing by controlling the false discovery rate using the Benjamini Hochberg correction and tested against the threshold p_BH_ < 0.05 for 100% of 50 bootstrap iterations (next paragraph). Significant diagnoses with undefined odds ratios in the logistic regression (e.g. Intestinal infection, OR = 1/∞, *p* = 0, n_indicated_preterm_ = 0, n_term_ = 30) were dropped.

As most of the diagnoses occur in fewer than 3% or 250 patients, we evaluated whether associations with each rare diagnosis were robust to small changes in the cohort. We retested all such associations 50 times removing one instance of each diagnosis. For each iteration, a unique individual was selected. If the number of instances of the diagnosis was less than 50, then each instance was removed in exactly one iteration. If the number of instances of the diagnosis was 50 or greater, then each instance was removed at most in one iteration.

**Fig. 2:**
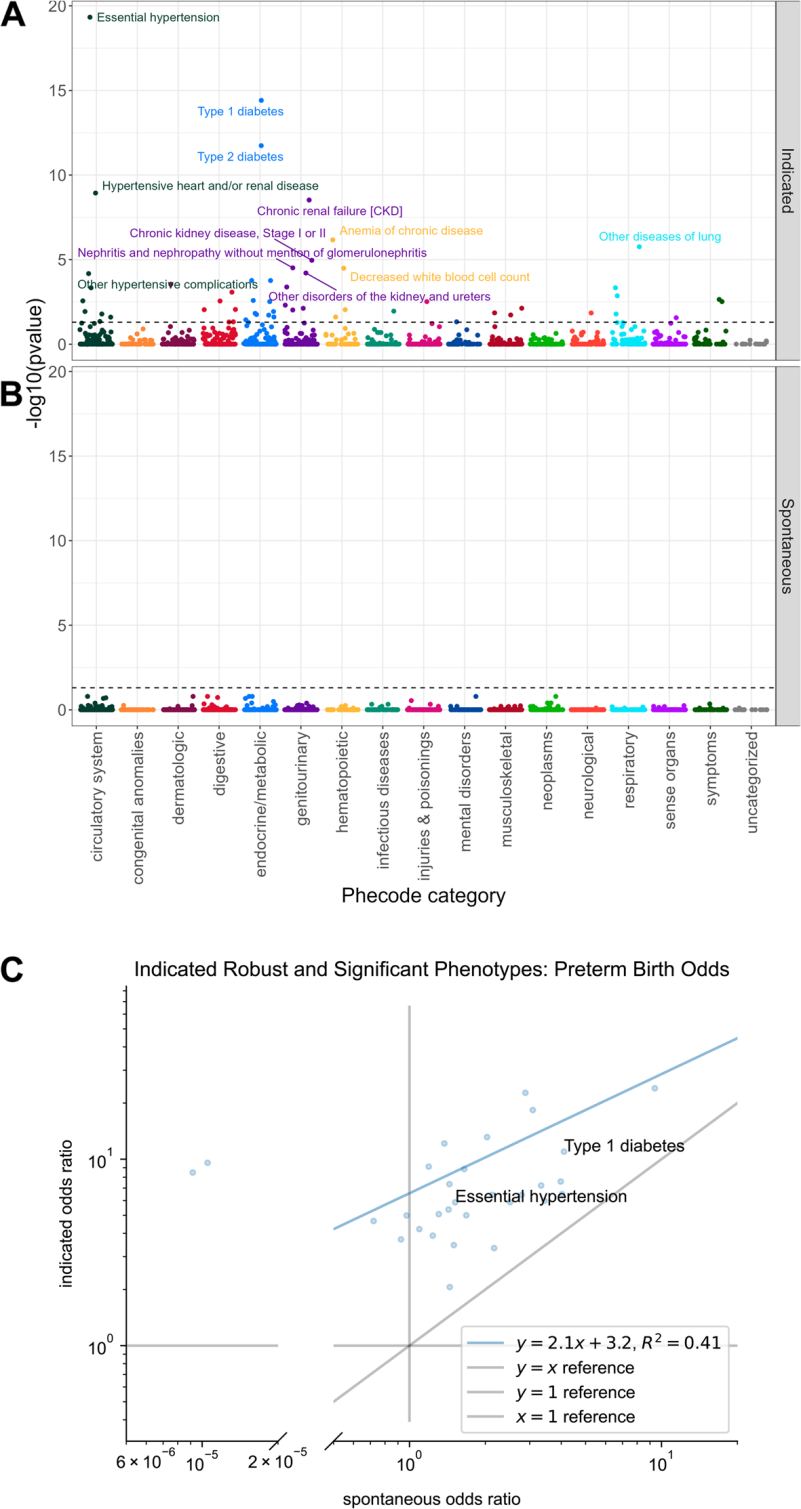
Many diagnoses associate with risk for indicated preterm birth, but none with spontaneous preterm birth. **A** *P*-values from logistic regression tests of the association of 1322 diagnoses with indicated preterm (*n* = 418) vs. term births (*n* = 9671). Thirty diagnoses passed the multiple testing correction at Benjamini–Hochberg FDR threshold of 5% (dashed line) and were robust to small changes in the data set. **B** *P*-values from logistic regression tests of the association of 1322 diagnoses with spontaneous preterm (*n* = 449) vs. term births (*n* = 9671). No diagnoses significantly associated with spontaneous preterm birth. **C** Comparison of the odds ratios for the 30 diagnoses significantly associated with indicated preterm birth between tests for indicated and spontaneous preterm birth. The odds ratios are correlated (*r*^2^ = 0.41, linear regression, left outliers dropped), but the relationships have systematically lower magnitude in the spontaneous cohort. The two most significant indicated diagnoses are labeled

Manhattan and forest plots were generated using the R packages ggplot2 (version 3.4.2) [33]​ and ggrepel (version 0.9.3) [34]​. Figures 2c, S1, and S3 were plotted using the Python packages Matplotlib (version 3.7.0) [35]​ and Seaborn (version 0.12.0) [36]​. Plots show covariate-adjusted *p*-values. Odds ratio 95% confidence intervals are not adjusted for multiple hypothesis testing.

Results tables for the overall and indicated analyses show the phecode, phenotype, OR (95% CI), and adjusted *p*-value for all phecodes that were significant in at least one iteration of the robustness analysis. For the spontaneous analysis, the tables show all results with an adjusted *p-*value < 0.5.

### Sensitivity analyses

The inclusion criteria for our main analysis require that a diagnosis exists prior to conception. To understand the impact of excluding individuals without pre-conception diagnoses, we repeated our analysis, including these individuals. We also repeated our analysis without any covariates.

## Results

### A densely phenotyped preterm birth cohort linked to electronic health records

To validate known clinical risk factors and identify potential new factors of PTB, we defined cohorts of preterm and term deliveries based on curated data from the UCSF Perinatal Database (PDB) and linked these to diagnoses from the UCSF electronic health record (EHR) database. The cohort consisted of 10,643 deliveries to 9,399 individuals from 2001 to 2022 (Fig. 1a). There were 975 (9.2%) PTBs in the cohort, which we further classified as spontaneous PTBs (*n* = 449, 4.2%) or indicated PTBs (*n* = 418, 3.9%). The remaining 108 (1.0%) PTBs could not be classified. Each of the preterm groups (spontaneous, indicated, all) was compared to term “controls” born at 37 weeks or later (*n* = 9671, 91%). More details about the cohorts are provided in the Methods section.

The demographics of the cohort reflected the population of the San Francisco Bay area served by UCSF. Most individuals had more than 12 years of education (84%). A large majority also used private insurance for the delivery (93%). The mean maternal age was 34 years, and maternal age ranged from 14 to 55 years. There were no significant differences in maternal age between indicated, spontaneous, and term individuals (Figure S1a; p_indicated-term_ = 0.2, p_spontaneous-term_ = 0.1, p_indicated-spontaneous_ = 0.9, Mann–Whitney U test). The two most represented self-reported racial categories were single-race white (48%) and single-race Asian/Pacific Islander (25%) (Table 1).

**Table 1:** Demographics

	Indicated Preterm	Spontaneous Preterm	All Preterm	Term	Overall
	(*N* = 418)	(*N* = 449)	(*N* = 973)	(*N* = 9671)	(*N* = 10,643)
**Race**					
Single race-White	171 (40.9%)	205 (45.7%)	426 (43.8%)	4715 (48.8%)	5141 (48.3%)
Single race-Black	55 (13.2%)	44 (9.8%)	108 (11.1%)	523 (5.4%)	631 (5.9%)
Single race-Latina	57 (13.6%)	46 (10.2%)	111 (11.4%)	679 (7.0%)	790 (7.4%)
Single race-Asian/Pacific Islander	79 (18.9%)	100 (22.3%)	200 (20.6%)	2466 (25.5%)	2666 (25.0%)
Multi race-Latina + other race	23 (5.5%)	17 (3.8%)	48 (4.9%)	433 (4.5%)	481 (4.5%)
Multi race-other races	10 (2.4%)	11 (2.4%)	23 (2.4%)	253 (2.6%)	276 (2.6%)
Other race	17 (4.1%)	17 (3.7%)	37 (3.8%)	440 (4.6%)	477 (4.5%)
Unknown	6 (1.4%)	9 (2.0%)	20 (2.1%)	162 (1.7%)	182 (1.7%)
**Maternal age (Years)**					
Mean (SD)	33.9 (6.1)	34.0 (5.4)	34.0 (5.64)	34.5 (4.84)	34.4 (4.92)
Median [Min, Max]	35.0 [15.0,51.0]	35.0 [14.0,55.0]	35.0 [15.0, 54.0]	35.0 [14.0, 55.0]	35.0 [14.0, 55.0]
Missing	0 (0%)	0 (0%)	0 (0%)	1 (0.0%)	1 (0.0%)
**Private Insurance**					
No	54 (12.9%)	40 (8.9%)	102 (10.5%)	579 (6.0%)	681 (6.4%)
Yes	363 (86.8%)	407 (90.6%)	865 (88.9%)	9050 (93.6%)	9915 (93.2%)
Unknown	1 (0.2%)	2 (0.4%)	6 (0.6%)	41 (0.4%)	47 (0.4%)
**Maternal education**					
< 12 years	13 (3.1%)	11 (2.4%)	25 (2.6%)	71 (0.7%)	96 (0.9%)
12 years	124 (29.7%)	76 (16.9%)	218 (22.4%)	1348 (13.9%)	1566 (14.7%)
> 12 years	230 (55.0%)	308 (68.6%)	589 (60.5%)	7550 (78.1%)	8139 (76.5%)
Unknown	51 (12.2%)	54 (12.0%)	141 (14.5%)	702 (7.3%)	843 (7.9%)
**Diagnosis count**					
Number of unique diagnoses Mean (SD)	10.9 (13.4)	8.8 (10.1)	9.6 (11.8)	7.3 (8.5)	7.5 (8.9)
Median [Min, Max]	6 [1,101]	5 [1,88]	5 [1.0,101]	5 [1,118]	5 [1,118]
**Diagnosis time**					
All visit times per individual, years before conception Mean (SD)	1.8 (1.6)	1.7 (1.6)	1.7 (1.6)	1.5 (1.6)	1.6 (1.6)
Median [Min, Max]	1.3 [0.0,8.7]	1.1 [0.0,9.0]	1.2 [0.0,9.0]	1.0 [0.0,21.7]	1.0 [0.0,21.7]
First visit time per individual, years before conception Mean (SD)	2.4 (1.9)	2.1 (1.9)	2.3 (1.9)	1.9 (1.8)	2.0 (1.9)
Median [Min, Max]	2.0 [0.0,8.7]	1.5 [0.0,9.0]	1.7 [0.0,9.0]	1.3 [0.0,21.7]	1.4 [0.0,21.7]

Race, maternal age, maternal education level, insurance status (private, public, or unknown), and diagnosis distributions of individuals included in this study. “Diagnosis Time” represents the number of years before conception that diagnoses occurred, which we assume to be predominantly during medical visits. For “All visit times per individual,” we included duplicate diagnoses over multiple visits for the same individual and excluded duplicate dates (i.e., multiple diagnoses on the same date for the same individual)

For each individual, we identified all diagnoses present in their EHR before conception (Fig. 1b). We harmonized diagnosis billing codes into phecodes, a curated grouping of ICD codes intended to capture clinically meaningful concepts (Figure S2). We identified 1,322 unique diagnosis phecodes in 18 organ systems across the cohort (Table S1), and individuals had an average of 8 unique diagnoses in their record prior to conception (Figure S1b). The top 5 diagnoses represented in our cohort (all births) are “Infertility, female” (*n* = 2,355), “Irregular menstrual cycle/bleeding” (2,149), “Abdominal pain” (1,657), “Miscarriage; stillbirth” (1,278), and “Pain in joint” (1,216). Most diagnoses (87%) were rare—occurring in fewer than 1% of patients (Figure S3a). Most medical visits with a diagnosis occurred within 2 years before conception (Figure S3b); over 95% of individuals’ EHR start date was less than 2.5 years before conception (Figure S1c); and the maximum EHR length was 21.7 years before conception (Figure S3b).

### Diverse pre-conception diagnoses associate with indicated PTB risk

We tested each of the 1,322 diagnoses present in the cohort for association with preterm vs. term birth using logistic regression with maternal age, maternal education, and insurance status, as covariates (Fig. 1c). Of the covariates, maternal education had the highest rate of missingness at 7.9%. Race was missing for 1.7% of the cohort, and insurance classification was missing for 0.4% of the cohort (Table 1). We adjusted for multiple testing by controlling the false discovery rate (FDR) at 5% using the Benjamini–Hochberg procedure and evaluated robustness of significant associations with an iterative data masking strategy (Methods and Fig. 1c).

**Fig. 3:**
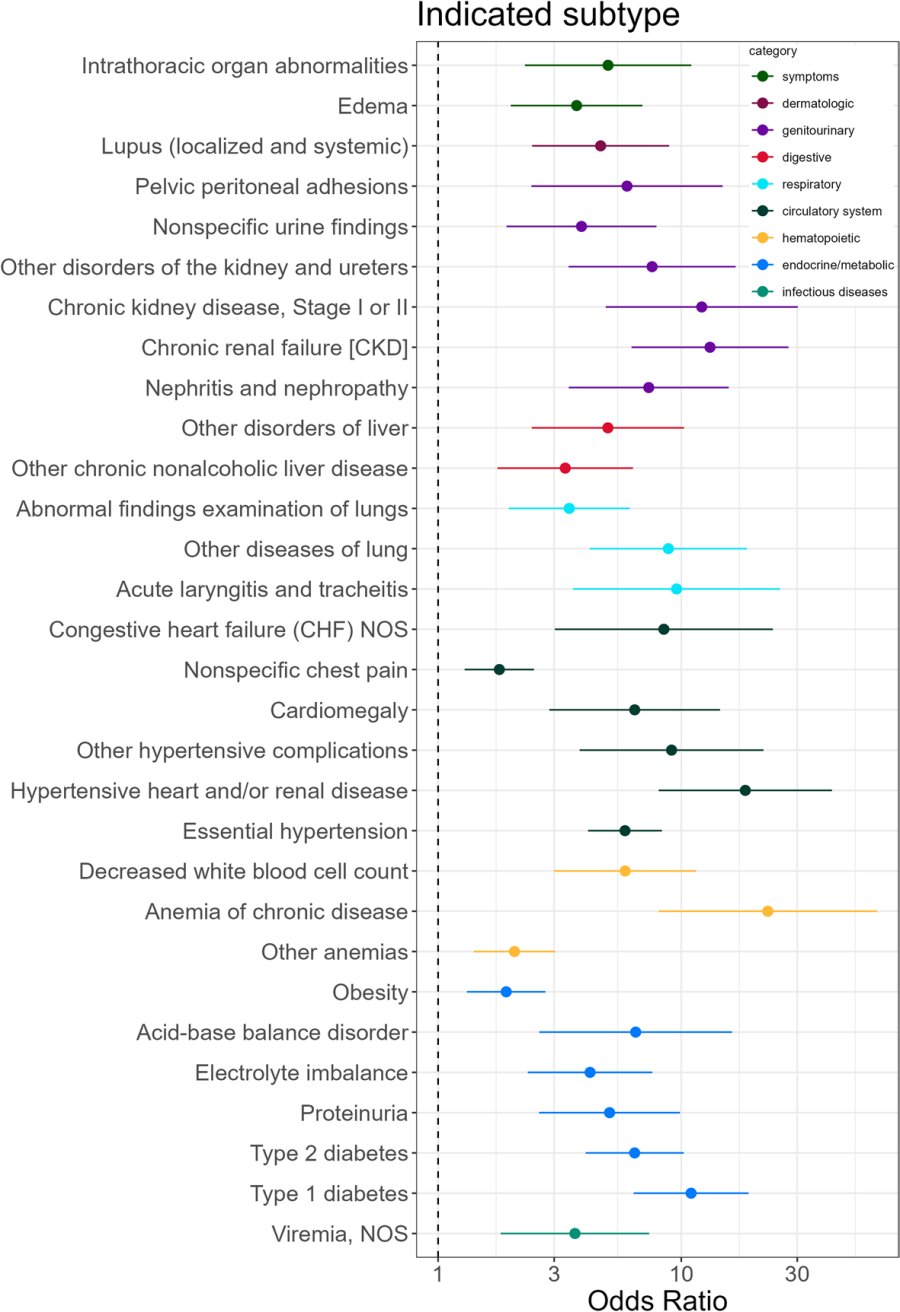
All significant and robust associations were associated with increased risk of indicated PTB. The odds ratios and confidence intervals for all significant phecode associations with indicated PTB. The associations spanned phecode categories (colors)

We identified 30 significant and robust indicated preterm birth associations among the 1,322 diagnoses tested in the logistic regression (Figs. 2a and 3). As expected, the most significant associations aligned with well-established risk factors and clinical guidelines for inducing early labor: essential hypertension (adjusted *P*_*BH*_ = 4 × 10^−20^, *OR* = 6 (95% CI 4-8)), type 1 diabetes (adjusted *P*_*BH*_ = 4 × 10^–15^*, OR* = 11 (95% CI 6-19)), and type 2 diabetes (adjusted *P*_*BH*_ = 1.8 × 10^−12^, *OR* = 6 (95% CI 4-10)).

After diabetes and hypertension-related diagnoses, chronic kidney disease (CKD) was the next strongest preterm birth association (adjusted *P*_*BH*_ = 3 × 10^−9^). Several other renal diagnoses were also among the significant associations, including a kidney replaced by transplant and other disorders of the kidney and ureters.

The remainder of the significant associations included blood disorders, cardiac conditions, pulmonary conditions, liver conditions, electrolyte imbalances, and digestive conditions. To explore the meaning of the unspecific diagnoses “Other disorders of liver” and “Other diseases of lung,” we extracted concepts from clinical notes using ctakes [18]. The “Other disorders of liver” (*n* = 55) diagnosis represents diagnoses including liver lesion (*n* = 20), liver cirrhosis (*n* = 15), liver mass (*n* = 15), liver carcinoma (*n* = 14), and fatty liver (*n* = 13). The “Other diseases of lung” (*n* = 38) diagnosis represents diagnoses including lung consolidation (*n* = 11), interstitial lung diseases (*n* = 5), and lung mass (*n* = 4). Odds ratios, *P* values, and sample sizes for associations between indicated PTB and all significant and robust diagnoses are in Table S2.

### No pre-conception diagnoses are associated with spontaneous preterm birth

Given the strong associations with indicated preterm birth, we next tested for associations between diagnoses and spontaneous preterm birth vs. term. In contrast to indicated PTB, no diagnoses were significantly associated with spontaneous preterm birth (Fig. 2b). The absence of significant associations with spontaneous preterm birth is not due to lower statistical power than for indicated preterm birth, given their similar sample size (*n*_*spontaneous*_ = 449, *n*_*indicated*_ = 418).

Of the 30 diagnoses associated with indicated preterm birth, 25 follow similar trends in spontaneous preterm birth, albeit at much lower effect sizes (r^2^ = 0.41, *linear regression*, Fig. 2c). For example, hypertension has an odds ratio of 6 for indicated and 1.5 for spontaneous. There are five diagnoses with different directions of effect, including acute laryngitis and tracheitis and congestive heart failure (CHF) not otherwise specified (NOS); these are significant, robust risk factors for indicated preterm birth but are in the protective direction (though not significant) for spontaneous preterm birth. Table S4 lists odds ratios, *p-*values, and sample sizes for associations between spontaneous PTB and all significant and robust diagnoses.

### Associations in the combined analysis are driven by the indicated associations

**Fig. 4:**
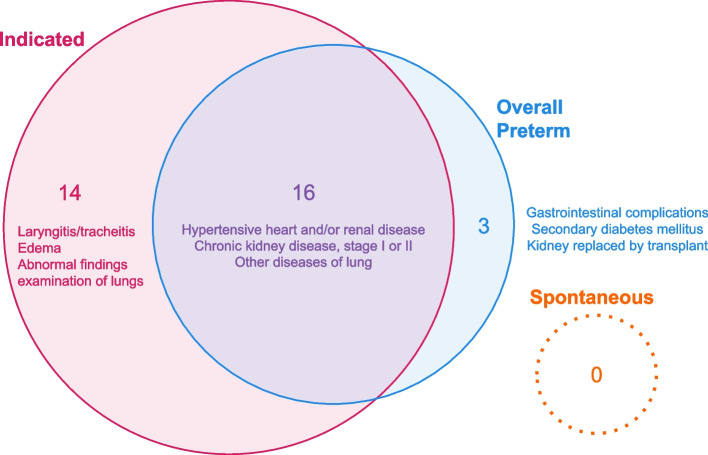
The strongest associations with overall preterm birth are also associated with indicated preterm birth, but not spontaneous preterm birth. Many kidney, cardiac, liver, and pulmonary conditions and diabetes are associated with overall and/or indicated preterm birth. Three risk factors for overall preterm birth were not discovered in the indicated preterm birth analysis

Next, we combined indicated and spontaneous PTBs and discovered 19 significant diagnoses associated with all PTB (Figure S4). Of these 19, 16 diagnoses were also significant in the indicated subgroup (Fig. 4). Diabetes, kidney diseases, and hypertension were the main diagnosis categories associated with indicated (Fig. 2a) and overall (Figure S4 and Table S3) PTB. The diagnoses associated with only indicated PTB but not the overall PTB cohort were spread across organs including the liver, lung, and heart. “Gastrointestinal complications,” “Secondary diabetes mellitus,” and “Kidney replaced by transplant” were significant and robust associations with overall PTB that were not found for indicated PTB.

### Associations with rare diagnoses are not robust

To address the potential impact of small sample size for rare diagnoses, we performed a robustness analysis, described under “*P*-Value Significance, Bootstrapping and Plotting” in the Methods section. Briefly, in each iteration of the analysis, a patient with the diagnosis was dropped from the population and the results were recomputed. Only diagnoses that remained significant in all 50 iterations of this analysis were considered significant and robust. This analysis removed several rare diagnoses with a small number of patients (e.g., “diabetic retinopathy” n_term_ = 1 n_indicated_ = 5). The rarest significant diagnosis that we report is “anemia of chronic disease,” n_term_ = 7 n_indicated_ = 9. The only other n_indicated_ < 10 or n_term_ < 10 significant diagnoses have at least 8 observations and are related to hypertension or renal disorders (Table S2), which are previously established indicators for indicated preterm birth.

### Our main conclusions are robust to cohort definition and consideration of covariates

One of the inclusion criteria for our cohort is that a diagnosis exists prior to conception. While this increases our confidence that the health history of each person is known, it may bias the cohort towards individuals with more prior conditions. To explore the sensitivity of our conclusions to the cohort definition, we repeated our analysis removing this inclusion criterion.

The sample size for this sensitivity analysis was 38,896 individuals with 2,048 indicated PTBs and 2,305 spontaneous PTBs. In the indicated subgroup, there were 17 significant associations, all of which were also significant in the main analysis of the indicated subgroup (Figure S6 and Table S6). Consistent with the main analysis, no significant associations were found for the spontaneous subgroup (Figure S6 and Table S7). In the overall group, eight diagnoses were significantly associated with PTB, all of which were also significant in the main analysis. The most significant associations were hypertension, type 1 diabetes, and type 2 diabetes (Figure S5 and Table S5).

In addition, we repeated our analysis without any covariates. As expected, this yielded more associations; in the indicated subgroup, 52 diagnoses were significantly associated with PTB (Table S8, Figure S7). Similar to the main analysis with covariates, the most significant diagnoses were hypertension, diabetes, and renal diseases. No diagnoses were significantly associated with spontaneous PTB in this analysis (Table S9). In the overall group, 45 diagnoses were significantly associated with PTB (Table S10 and Figure S8). In both the indicated and overall groups, all diagnoses that were significant in the main analysis adjusting for covariates were also significant in this analysis without covariates.

## Discussion

Our study uses the rich phenotype data present in EHRs to generate hypotheses about the connection between pre-conception diagnoses and risk for indicated and spontaneous PTB. Using a densely phenotyped cohort with curated delivery details enabled us to investigate 1322 diagnoses across 18 different phecode categories. In our analysis of indicated PTB, we replicated known associations, including hypertension, diabetes, and chronic kidney disease. We also found several associations that warrant further investigation. By contrast, we found no associations between preconception diagnoses and spontaneous PTB. This underscores the limitations of approaches that do not differentiate between indicated and spontaneous preterm birth.

The most significant hits of our study replicated well-established risk factors for PTB, with the four most significant being type 1 diabetes, essential hypertension, type 2 diabetes, and hypertensive heart and/or renal disease. These likely reflect clinical practice as they have existing recommendations for preterm delivery [9]. Additionally, several significant diagnoses relate to kidney function, such as chronic kidney disease, chronic renal failure, and other disorders of the kidney and ureters. Associations between preterm birth and CKD have been observed in studies around the world, but the mechanisms and relevance to risk are not well understood [19]. Harel et. al propose that pre-pregnancy counseling, increased monitoring of the mother and fetus, and aspirin treatment to prevent preeclampsia would likely improve pregnancy outcomes for mothers with CKD, as indications for delivery are often hypertensive disorders, worsening renal function, fetal growth restriction, abnormal antenatal testing, or worsening maternal morbidity [19].

We found an association between decreased white blood cell count and overall PTB (Figure S4a and S4b). However, this relationship is inconsistent in the literature; some studies found no association [20] while others found support for this association [21, 22]. The connection between PTB and pre-conception lung conditions, including lung consolidation, interstitial lung diseases, and lung mass, is not well studied. Preterm birth causes lung conditions in the newborn through adulthood [23], and preterm birth risk is heritable in families [24]. This could contribute to the association observed between PTB and pre-conception lung diagnoses. Furthermore, we observed associations between PTB and liver conditions, including liver lesions, liver cirrhosis, liver mass, liver carcinoma, and fatty liver. Connections between liver dysfunction and PTB have also been previously identified in more targeted studies [25–27].

Another major strength of our study is our use of a physician-curated births database to differentiate between spontaneous and indicated PTBs. This information cannot be reliably extracted from most EHR data. Prior work has identified outcome misclassification as a concern for such EHR-based association studies [28]. Additionally, researchers investigating obstetric data quality in an EHR system found that quality was varied and recommended manual abstraction where possible [29]. By using a database reviewed by physicians to define our outcome, we minimize the risk of misclassification.

We found multiple diagnoses associated with indicated PTB, but none associated with spontaneous PTB. This pattern is likely explained by the fact that established risk factors are key to clinicians’ decision-making when it comes to indicating delivery. When combined, significant associations were entirely driven by indicated PTBs, even though the spontaneous and indicated groups were of a similar size. Thus, our understanding of risk factors for spontaneous PTB remains limited.

There are several limitations in our study. Our data come from a tertiary care center. Thus, the patients and deliveries seen at this facility are not representative of the overall local population, and many patients seen for delivery do not have a previous clinical record at the facility. We present both patient demographics (Table 1) and sample selection (Fig. 1) to show the context in which the study was performed. Compared to the state of California, the PTB rate among our cohort is similar; however, our study population was generally older, more educated, and was privately insured at a high rate. The absence of a significant difference in age distribution between our indicated, spontaneous, and term cohorts may not reproduce in a US age-representative dataset (Figure S1a).

Major chronic health condition diagnoses are common in our cohort, yielding strong statistical power. While we captured diagnoses across all major medical specialties using EHRs, some diagnoses are under recorded and others are rare. Consequently, we expected and found low sample size and weak statistical power in our cohort for many diagnoses. More specifically, there are over 585 diagnoses that occur in fewer than 10 individuals (Figure S3a), and over 300 of these are not present in any individuals who delivered preterm.

We expected that most diagnoses would be recorded in the short time before conception, and we found that over 50% of diagnoses occurred within 1 year of conception (Figure S3b). This represents a common limitation of EHR trajectory analysis research: patients often use many healthcare institutions over their lifetime, and a patient’s medical history at any individual institution is usually missing data from previous institutions. This may also explain the reason our sensitivity analysis found fewer associations – while we increased our sample size, we likely introduced exposure misclassification (i.e., an incorrect assumption that the absence of a recorded diagnosis means that an individual does not have that diagnosis).

Medical practice changes over time, and this affects diagnosis classifications and diagnosis generality. Several relevant medical practices have changed in the time covered by our cohort—birth dates 2001–2022. For example, the American College of Cardiology and American Heart Association created a lower blood pressure definition of hypertension in 2017 [30], and hundreds of new ICD-10 codes are created each year [31]. These changes, among others, affect diagnosis consistency between years, but we anticipate that such changes would largely decrease power rather than lead to spurious associations.

Furthermore, it can be challenging to interpret associations given the lack of specificity of some diagnoses. For example, “Abnormal findings examination of lungs” is a very general concept and can represent a range of undiagnosed diseases and underlying conditions. Additionally, the diagnoses codes do not fully capture the severity of disease: a diagnosis of alcoholism does not distinguish mild or moderate alcohol consumption; a diagnosis of type 2 diabetes applies to both well-managed and poorly managed disease. Nonetheless, in the poorly managed case, we are likely to see additional diagnoses representing additional complications. Future studies that supplement diagnosis data with severity estimates could provide further insights about the relationships between disease severity and PTB. In EHR studies, this could be supplemented with lab, vitals, and/or clinical notes data.

Our study only investigated one stratification of PTB—spontaneous/indicated—and one gestational age threshold. We ran preliminary studies on stratifying early (< 32 weeks gestational age) and late (32–36 weeks) PTB, but the sample size for the early preterm group (*n* = 132) was too small to produce any meaningful results on potential diagnosis associations. Future work should explore associations for different stratifications such as early/late preterm, young/mid/old maternal age, rural/suburban/urban maternal home, and low/middle/high maternal socioeconomic status. Identifying different risk factors and pathways for different subtypes of PTB could lead to a better understanding of this heterogeneous condition and to targeted and effective prevention efforts.

## Conclusions

Our study demonstrates that analyses that combine spontaneous and indicated PTB will have shortcomings. Due to established clinical practices and the apparent heterogeneity of spontaneous PTB, the indicated group will have higher rates of known risk factors and thus have an outsized effect on combined analyses. Further investigation focused on the spontaneous PTB population is imperative since it has the potential to uncover novel associations that may help reveal new pathways and understanding of PTB.

We propose two main areas of further research resulting from this work. The first is investigating the lesser-known and new associations from our overall preterm analysis. Our work suggests several hypotheses that warrant more detailed study, especially in EHR systems complemented by other data sets. For instance, further work investigating the gastrointestinal associations would benefit from a combined EHR and gut microbiome cohort. The second area we suggest for future work is a thorough investigation into spontaneous PTB in this and other cohorts. We found no associations in this group, suggesting that larger sample sizes and alternative approaches are required. We propose that dimensionality reduction and clustering techniques may help identify subtypes in this heterogeneous phenotype. We are also eager to study events and exposures during the pregnancies and self-reported medical history at delivery.

## Supplementary Information

Supplementary Material 1: Table S1: Distribution of Diagnoses by Phecode Category.

Supplementary Material 2: Table S2: Many Diagnoses are Significantly and Robustly Associated with Indicated PTBs vs. Term Births. The most significant diagnoses' odds ratios, *p*-values, robustness percentages, and number of individuals are listed.

Supplementary Material 3: Table S3: Many Diagnoses are Significantly and Robustly Associated with All PTBs vs. Term Births. The most significant diagnoses' odds ratios, *p*-values, robustness percentages, and number of individuals are listed.

Supplementary Material 4: Table S4: No Diagnoses are Significantly Associated with Spontaneous PTBs vs. Term Births. The most significant (adjusted *p*-value < 0.5) diagnoses' odds ratios, *p*-values, and number of individuals are listed.

Supplementary Material 5: Table S5: Many Diagnoses are Significantly and Robustly Associated with All PTBs vs. Term Births, After Including Individuals with No Pre-Conception Diagnoses. The most significant diagnoses' odds ratios, *p*-values, robustness percentages, and number of individuals are listed.

Supplementary Material 6: Table S6: Many Diagnoses are Significantly and Robustly Associated with Indicated PTBs vs. Term Births, After Including Individuals with No Pre-Conception Diagnoses. The most significant diagnoses' odds ratios, *p*-values, robustness percentages, and number of individuals are listed.

Supplementary Material 7: Table S7: No Diagnoses are Significantly Associated with Spontaneous PTBs vs. Term Births, After Including Individuals with No Pre-Conception Diagnoses. The most significant (adjusted *p*-value < 0.5) diagnoses' odds ratios, *p*-values, and number of individuals are listed.

Supplementary Material 8: Table S8: Removing Covariates from our Analysis Results in More Associations between Diagnoses and Indicated PTBs. vs. Term Births The most significant diagnoses' odds ratios, *p*-values, robustness percentages, and number of individuals are listed.

Supplementary Material 9: Table S9: No Diagnoses are Significantly Associated with Spontaneous PTBs vs. Term Births, After Removing Covariates. The most significant (adjusted *p*-value < 0.5) diagnoses' odds ratios, *p*-values, and number of individuals are listed.

Supplementary Material 10: Table S10: Removing Covariates from our Analysis Results in More Associations between Diagnoses and All PTBs vs. Term Births.The most significant diagnoses' odds ratios, *p*-values, robustness percentages, and number of individuals are listed.

Supplementary Material 11: Figure S1: Patient Distributions for Maternal Age, Number of Diagnoses per Patient, First Visit per Patient, All Visits per Patient. (A) By 2-sided Mann–Whitney U rank test, there are no significant differences in maternal age between indicated, spontaneous, and term. (B) Additionally, by the same statistics test, the spontaneous preterm and indicated preterm patients have significantly more diagnoses than the term patients. Outliers dropped in visualization. (C) Indicated individuals have the longest (time) EHR length, followed by spontaneous individuals, then term individuals with the lowest. Outliers dropped in visualization.

Supplementary Material 12: Figure S2: Phecode Subgroups Used to Define Diagnoses. We use phecode subgroups to define diagnoses. This allows us to test diagnosis-preterm associations with sufficient detail.

Supplementary Material 13: Figure S3: Diagnoses Frequency Distribution. (A) Most (1,148 of 1,322) diagnoses occur rarely (in fewer than 100/10642 patients). (B) Most medical visits with a diagnosis occurred within 2 years before conception.

Supplementary Material 14: Figure S4: Testing Associations Between Diagnoses and Overall PTB Identifies Known Risk Factors and Novel Candidates. (A) *P* -values from logistic regression tests of the association of 1322 diagnoses with preterm (*n* = 973) vs. term births (*n* = 9671). Nineteen diagnoses passed the Benjamini Hochberg multiple testing corrected false discovery rate threshold of 5% (dashed line) and were robust to small changes in the data set. Diagnoses were represented as phecodes and plotted by phecode category. Significant, robust associations are labeled. (B) The forest plot shows odds ratios and 95% confidence intervals of the 19 diagnoses that were significantly and robustly associated with overall PTB. “Other disorders of liver” represents conditions including liver lesion, liver cirrhosis, liver mass, liver carcinoma, and fatty liver. “Other diseases of lung” represents conditions including lung consolidation, interstitial lung diseases, and lung mass.

Supplementary Material 15: Figure S5: Including Individuals without a Diagnosis Results in Similar Associations Between Diagnoses and Overall PTB. (A) *P*-values from logistic regression tests of the association of 1322 diagnoses with preterm (*n* = 4,353) vs. term births (*n* = 35,543). Nine diagnoses passed the Benjamini Hochberg multiple testing corrected false discovery rate threshold of 5% (dashed line) and were robust to small changes in the data set. Diagnoses were represented as phecodes and plotted by phecode category. Significant, robust associations are labeled. (B) The forest plot shows odds ratios and 95% confidence intervals of the 8 diagnoses that were significantly and robustly associated with overall PTB.

Supplementary Material 16: Figure S6: Including Individuals without a Diagnosis Results in Similar Associations Between Diagnoses and Indicated vs. Spontaneous PTB. (A) *P*-values from logistic regression tests of the association of 1322 diagnoses with indicated preterm (*n* = 2,048) vs. term births (*n* = 35,543). Twenty diagnoses passed the multiple testing corrected Benjamini–Hochberg threshold at FDR of 5% (dashed line) and were robust to small changes in the data set. (B) *P*-values from logistic regression tests of the association of 1322 diagnoses with spontaneous preterm (*n* = 2,305) vs. term births (*n* = 35,543). Consistent with the main analysis, no diagnoses significantly associated with spontaneous preterm birth. (C) The forest plot shows odds ratios and 95% confidence intervals of the 17 diagnoses that were significantly and robustly associated with indicated PTB.

Supplementary Material 17: Figure S7: Removing Covariates from our Analysis Results in More Associations between Diagnoses and Indicated PTB. *P*-values from logistic regression tests of the associations of 1322 diagnoses with indicated preterm (*n* = 418) vs. term births (*n* = 9671). 93 diagnoses were significant and robust. The most significant associations are labelled.

Supplementary Material 18: Figure S8: Removing Covariates from our Analysis Results in More Associations between Diagnoses and Overall PTB. *P*-values from logistic regression tests of the association of 1322 diagnoses with preterm (*n* = 973) vs. term births (*n* = 9671). 45 diagnoses were significant and robust. The most significant associations are labelled.

## Data Availability

The raw data that support the findings of this study are not publicly available due to patient privacy. Overall preterm, indicated preterm, and spontaneous preterm diagnosis association results are in Tables S2–S4. In our association analyses, some diagnoses had very low (< 10) patient counts. To maintain patient de-identification, exact counts, odds ratios, and p-values are redacted for those diagnoses in Tables S2–S4. UCSF-affiliated individuals can request access to UCSF EHR data by contacting UCSF Information Commons (Info.Commons@ucsf.edu). The custom code/software we generated are available in the repository “stratified_PTB_association_study” available here [https://github.com/hanmochturt/stratified_PTB_association_study]. This contains instructions for OMOP EHR data queries and all the code for patient filtering, diagnosis aggregation, overall PTB association analysis, indicated and spontaneous PTB association analysis, robustness testing, and figure creation.

## Abbreviations

EHR: Electronic Health Record
ICD: International Classification of Diseases
PTB: Preterm Birth
SES: Socioeconomic status
UCSF: University of California, San Francisco
