# Supplementary material for: Pre-conception clinical risk factors differ between spontaneous and indicated preterm birth in a densely phenotyped EHR cohort
Source: BMC Pregnancy Childbirth. 2025 Feb 12;25:149. doi: 10.1186/s12884-025-07166-2 (PMC11817080; doi:10.1186/s12884-025-07166-2)
Supplement: Supplementary file 1 — Supplementary Material 1: Table S1: Distribution of Diagnoses by Phecode Category. [file 12884_2025_7166_MOESM1_ESM.docx]

| Phecode category | Count of diagnoses |
| --- | --- |
| digestive | 125 |
| genitourinary | 111 |
| circulatory system | 108 |
| sense organs | 103 |
| endocrine/metabolic | 99 |
| musculoskeletal | 97 |
| injuries & poisonings | 93 |
| dermatologic | 80 |
| neoplasms | 79 |
| respiratory | 70 |
| neurological | 68 |
| mental disorders | 54 |
| infectious diseases | 52 |
| pregnancy complications | 49 |
| symptoms | 43 |
| hematopoietic | 38 |
| congenital anomalies | 36 |
| uncategorized | 17 |

**Table S1: Distribution of Diagnoses by Phecode Category**
