# Supplementary figures and images for: Pre-conception clinical risk factors differ between spontaneous and indicated preterm birth in a densely phenotyped EHR cohort
Source: BMC Pregnancy Childbirth. 2025 Feb 12;25:149. doi: 10.1186/s12884-025-07166-2 (PMC11817080; doi:10.1186/s12884-025-07166-2)

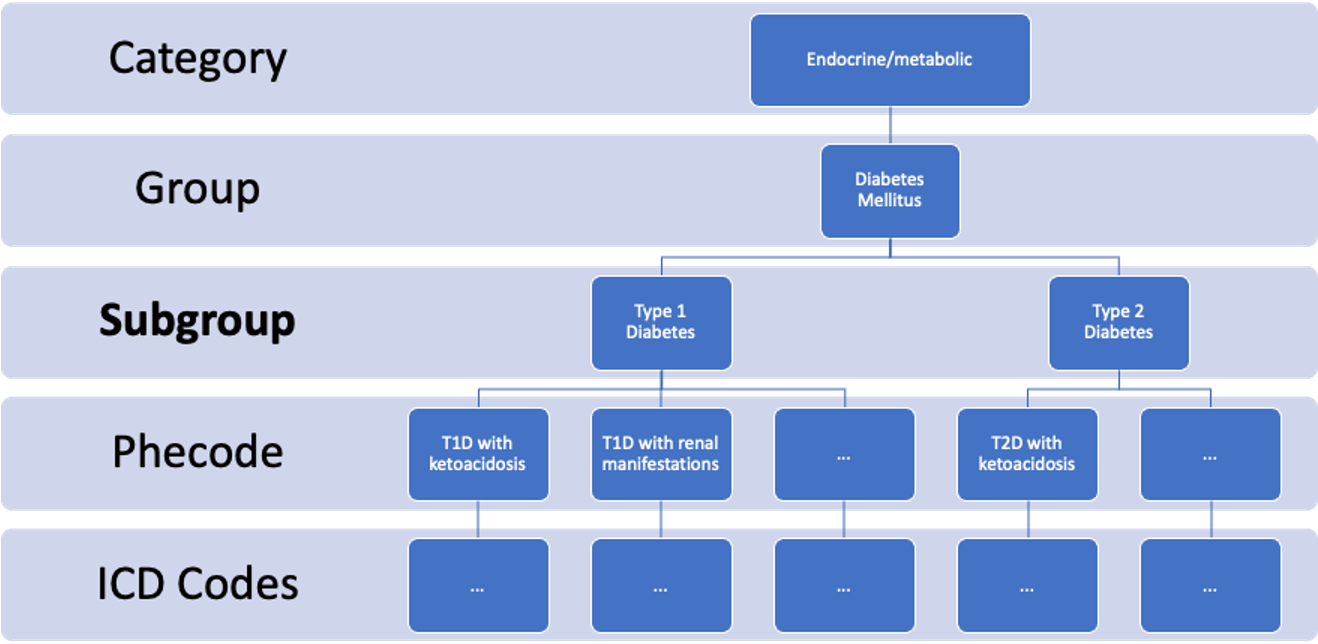

Supplement: Supplementary file 12 — Supplementary Material 12: Figure S2: Phecode Subgroups Used to Define Diagnoses. We use phecode subgroups to define diagnoses. This allows us to test diagnosis-preterm associations with sufficient detail. [file 12884_2025_7166_MOESM12_ESM.png]

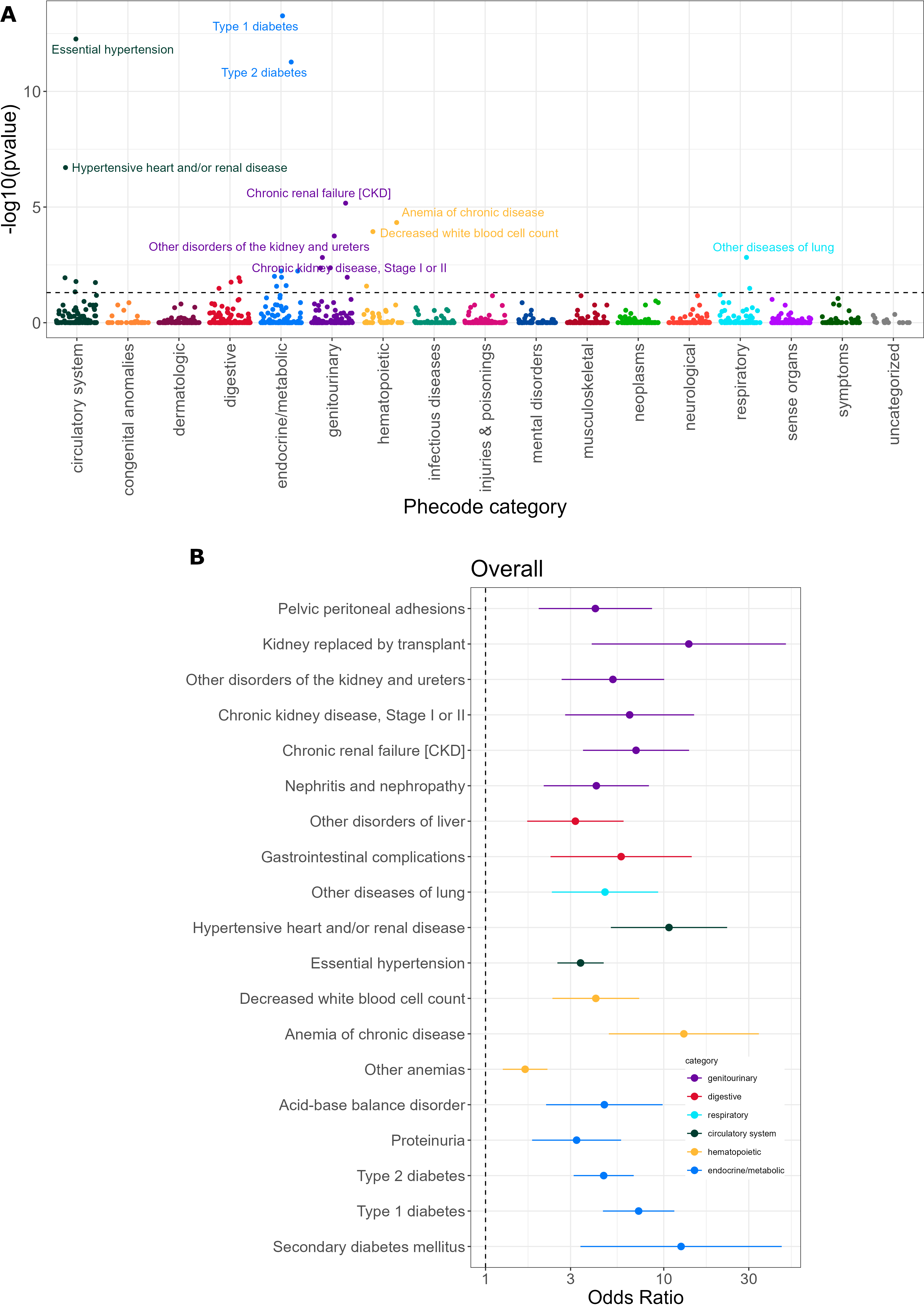

Supplement: Supplementary file 14 — Supplementary Material 14: Figure S4: Testing Associations Between Diagnoses and Overall PTB Identifies Known Risk Factors and Novel Candidates. (A) P -values from logistic regression tests of the association of 1322 diagnoses with preterm (n = 973) vs. term births (n = 9671). Nineteen diagnoses passed the Benjamini Hochberg multiple testing corrected false discovery rate threshold of 5% (dashed line) and were robust to small changes in the data set. Diagnoses were represented as phecodes and plotted by phecode category. Significant, robust associations are labeled. (B) The forest plot shows odds ratios and 95% confidence intervals of the 19 diagnoses that were significantly and robustly associated with overall PTB. “Other disorders of liver” represents conditions including liver lesion, liver cirrhosis, liver mass, liver carcinoma, and fatty liver. “Other diseases of lung” represents conditions including lung consolidation, interstitial lung diseases, and lung mass. [file 12884_2025_7166_MOESM14_ESM.png]

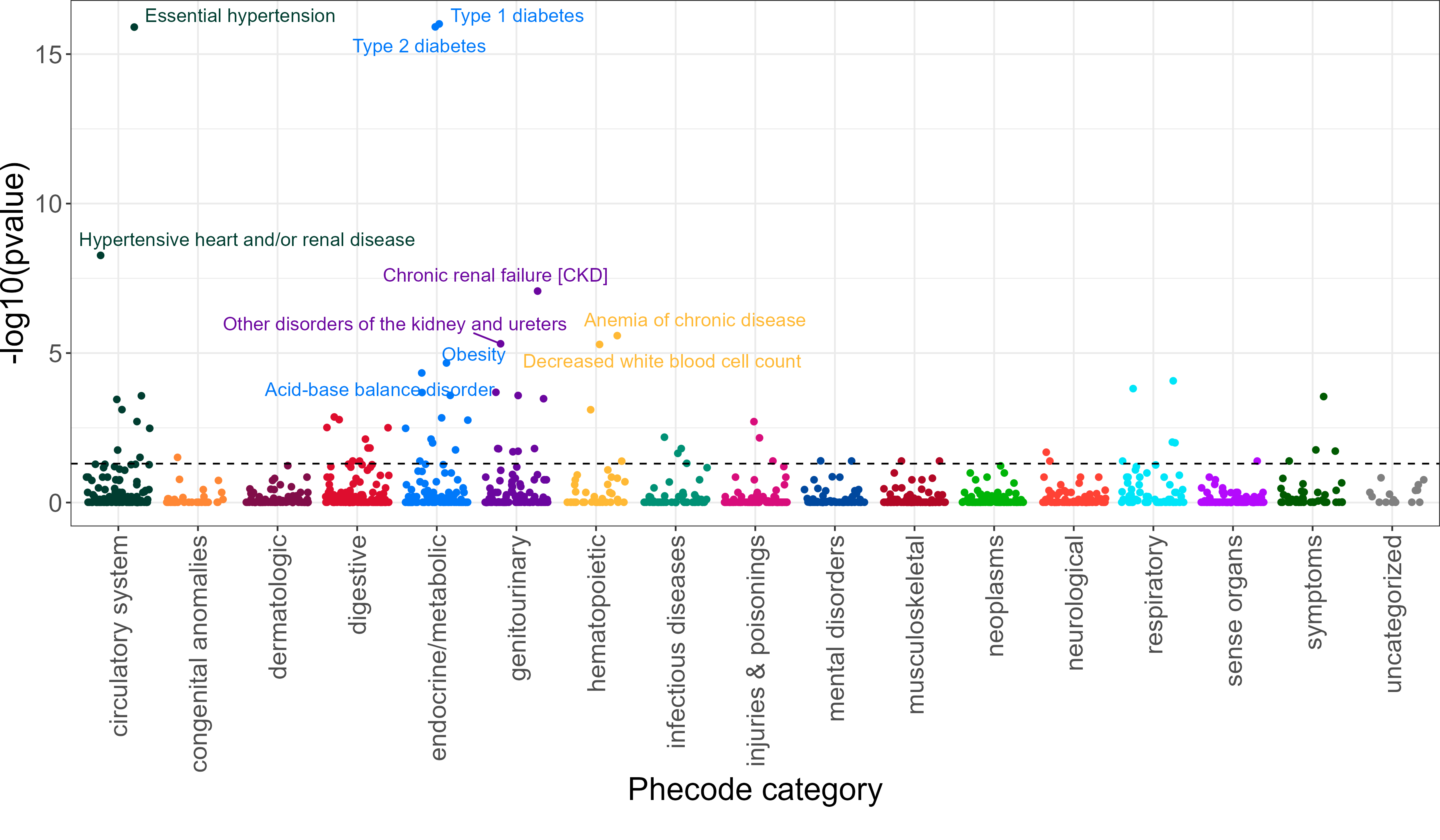

Supplement: Supplementary file 17 — Supplementary Material 17: Figure S7: Removing Covariates from our Analysis Results in More Associations between Diagnoses and Indicated PTB. P-values from logistic regression tests of the associations of 1322 diagnoses with indicated preterm (n = 418) vs. term births (n = 9671). 93 diagnoses were significant and robust. The most significant associations are labelled. [file 12884_2025_7166_MOESM17_ESM.png]

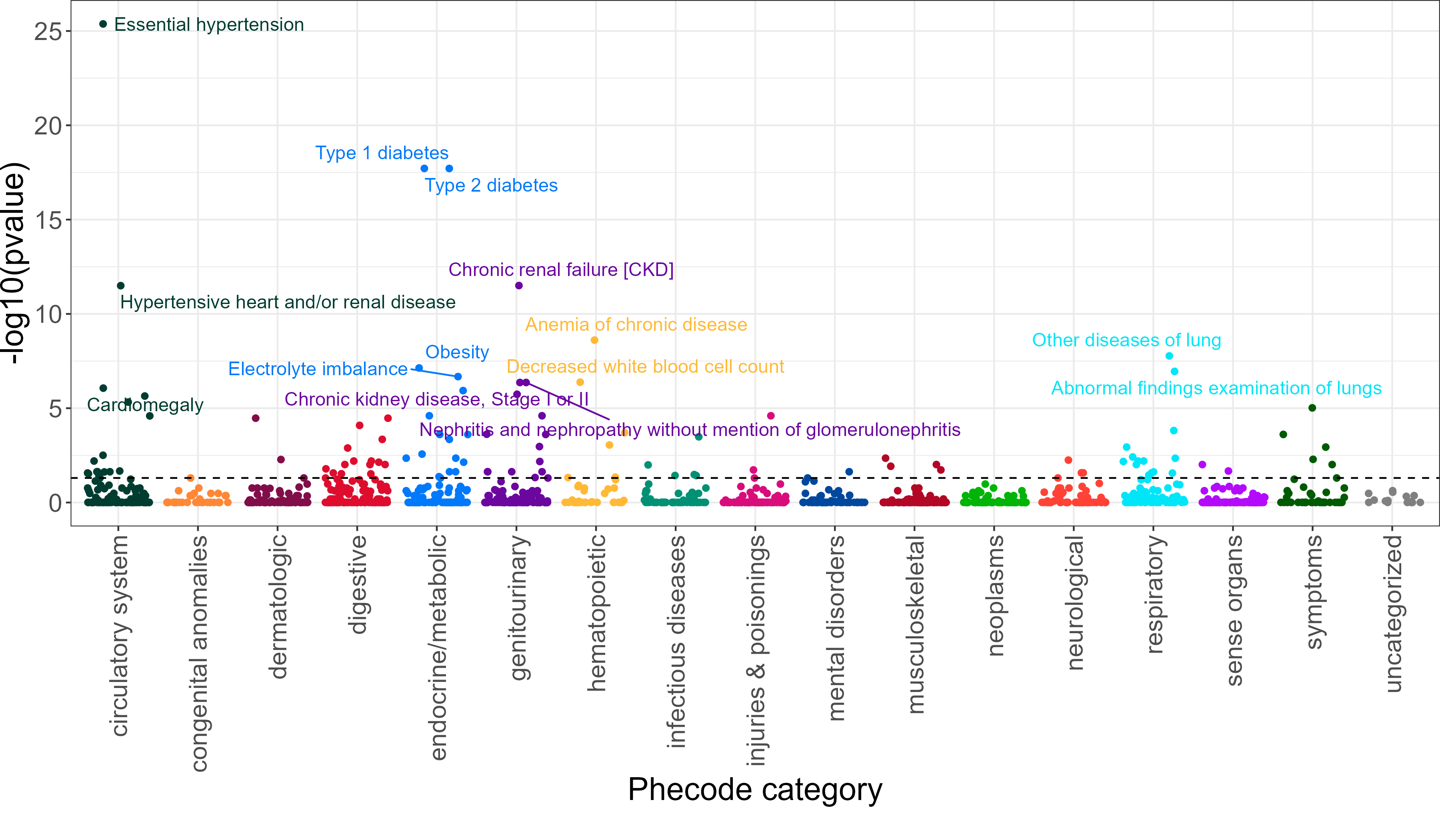

Supplement: Supplementary file 18 — Supplementary Material 18: Figure S8: Removing Covariates from our Analysis Results in More Associations between Diagnoses and Overall PTB. P-values from logistic regression tests of the association of 1322 diagnoses with preterm (n = 973) vs. term births (n = 9671). 45 diagnoses were significant and robust. The most significant associations are labelled. [file 12884_2025_7166_MOESM18_ESM.png]
